# Necrotizing Fasciitis of the Chest in a Neonate in Southern Nigeria 

**DOI:** 10.1155/2014/818059

**Published:** 2014-12-30

**Authors:** Oluwafemi Olasupo Awe, Emeka B. Kesieme, Babatunde Kayode-Adedeji, Quinzy O. Aigbonoga

**Affiliations:** ^1^Plastic Surgery Unit, Department of Surgery, Irrua Specialist Teaching Hospital, Irrua, Edo State, Nigeria; ^2^Plastic Surgery Unit, Department of Surgery, Ambrose Alli University, PMB 08, Ekpoma, Edo State, Nigeria; ^3^Cardiothoracic Surgery Unit, Department of Surgery, Irrua Specialist Teaching Hospital, Irrua, Edo State, Nigeria; ^4^Special Care Baby Unit, Department of Paediatrics, Irrua Specialist Teaching Hospital, Irrua, Edo State, Nigeria

## Abstract

We discuss the successful saving of a male neonate with necrotizing fasciitis of the chest following a hot fomentation of the umbilicus with exposure of the ribs and the pleural space on the right side. He recovered 5 weeks after admission. We stressed the need to recognize necrotizing fasciitis extending from the upper anterior abdominal wall to the chest following hot fomentation of the umbilicus. The need for multidisciplinary cooperation for excellent outcome is very important, that is, neonatologist, medical microbiologist, and plastic and chest surgeons.

## 1. Introduction

Necrotizing fasciitis (NF) is a life-threatening infection and necrosis of the skin, subcutaneous tissue, deep fascia, and sometimes underlying muscles, with a fulminant course and a high mortality rate. NF can be a complication of minor soft tissue infection, or it can occur after a trauma or surgical procedure. It is commoner in the lower extremities, perineum, and lower anterior abdominal wall but rare in retroperitoneal space, the chest, neck, and the scalp. Much more rare is necrotizing fasciitis in a neonate. We report a case of a male neonate with necrotizing fasciitis of the chest and the upper anterior abdominal wall. The importance of early aggressive serial surgical debridement, repeated sterile dressing, and antibiotics in the control of the disease is highlighted.

## 2. Case Report

A week-old male neonate was admitted via the paediatric emergency unit with complaint of swelling and discolouration of the chest and upper abdomen, excessive crying, and fever of 2-day history. The swelling was first noticed at the umbilicus but spread to the chest within few hours, and the swelling was associated with reddish discolouration of the overlying skin. The child was crying excessively particularly when the abdomen or the chest was touched.

There was 2-day antecedent history of hot water massage of the umbilicus by the grandmother before the onset of the symptoms. No other risk factor for sepsis was identified.

On examination, he was a male neonate, was febrile, and was anicteric. Pitting edema was over the chest and anterior abdominal wall extending from the clavicles superiorly, midaxillary lines laterally, and umbilicus inferiorly. There are one (1) necrotic area over the midsternum (2 cm × 4 cm) and two (2) on the right chest (about 2 cm × 2 cm each). The swelling was hyperaemic and tender. He was tachypneic (88 cpm) and dyspneic. Also he had tachycardia (186 bpm). Diagnosis of necrotizing fasciitis (NF) of the chest and upper anterior abdominal wall was made.

He was admitted to the Special Baby Care Unit and comanaged with both plastic and cardiothoracic surgeons. Blood culture and wound biopsy report yielded coagulase negative* Staphylococcus aureus* sensitive to azithromycin, erythromycin, and gentamicin. He had 4 spikes of fever and hypothermia in the first week of admission. The complete blood count on admission was packed cell volume 47%, white blood cell count of 7200/cmm, with relative neutropenia (30%). Electrolyte and urea results were normal.

**Figure 1:**
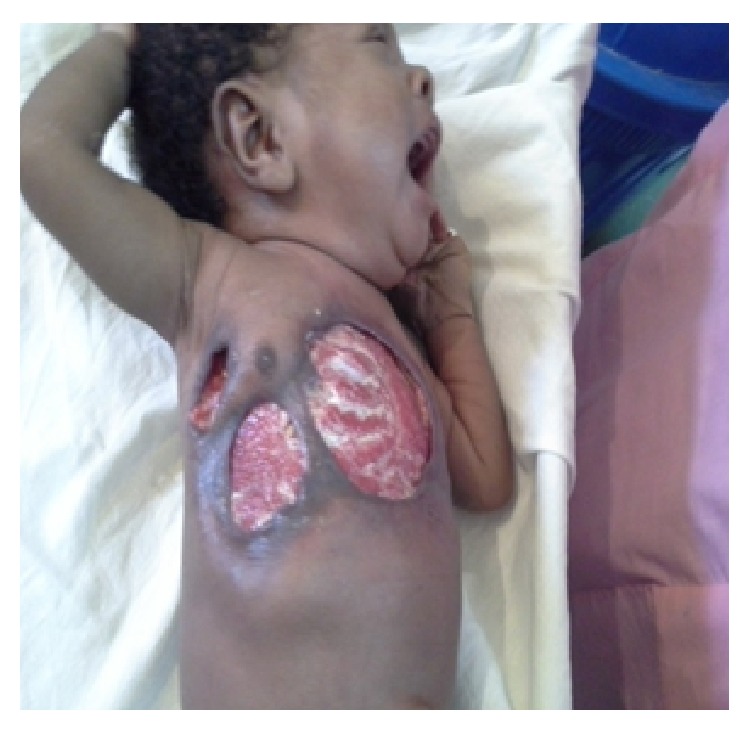
Immediate post-op.

Following resuscitation, packed cell volume was 27% and maternal retroviral screen was negative. C-reactive protein was not done because it is not available in the hospital. Empirical antibiotics were I.V. imipenem, metronidazole, Gentamycin, and I.M. ibuprofen. He had two (2) aliquots of blood transfused and serial wound debridement resulting in three (3) wounds interspersed by 3 cm wide skin tags. The wounds were 6 cm × 6 cm and two of 3 cm × 4 cm each over the sternum and right chest, respectively, with raised edge, granulating base exposing 3rd to 7th ribs, and pleural cavity (Figure 1). He had regular dressing with 1% povidone iodine and diluted honey. He was discharged home to continue dressing at the outpatient clinic on the 42nd day of admission when the wound was almost completely healed.

**Figure 2:**
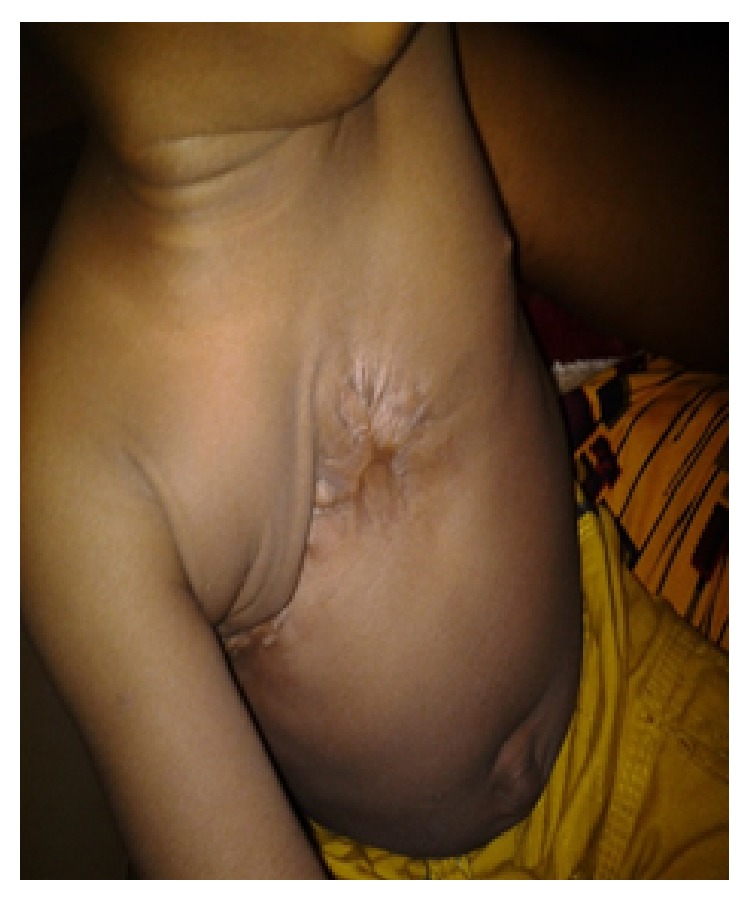
10 months after discharge.

He attended clinic twice over the next 3 months and 10 months thereafter for scar treatment (Figure 2).

## 3. Discussion

Necrotizing fasciitis is a severe soft tissue infection associated with rapidly progressive necrosis of the subcutaneous tissue and superficial fascia [1]. The disease is also characterized by early development of systemic toxicity [2]. NF is infrequent and is usually fatal in infants and children. There are reports of childhood NF resulting from appendicitis, intra-abdominal abscess, omphalitis, balanitis, and mammitis. Also predisposing factors vary with age, diabetes, and immune-suppressed status [3–6]. The NF of the chest wall has been secondary to some form of trauma, tumor resection, irradiation, or surgical procedure especially in the adults. There have been reported cases related to chest catheter drainage [7].

The early diagnosis of NF is very important in the management. The clinical symptoms and signs, such as erythematous rashes and other signs of sepsis, are important for differential diagnosis. In many cases, it is very difficult to distinguish early NF from cellulitis since fever, skin rashes, and other clinical findings are common symptoms of infection and sepsis.

The recent clinical classification of NF is distinguished into four types: NF type I (polymicrobial/synergistic, 70–80%), NF type II (20% of cases, usually monomicrobial), NF type III (Gram-negative monomicrobial, including marine-related organisms), and NF type IV (fungal) [8].

Our patient is a neonate who presented with most likely omphalitis being the leading point, though the eventual ulcers were closely related to the right nipple. It seems to be a monomicrobial NF with* Staphylococcus aureus*. The monomicrobial NF is common in patients with immunosuppression. The neonate is considered to be immune-suppressed because his immune system is not yet well developed [3, 4].

Aggressive surgery and debridement are usually required in combination with antibiotic therapy to limit the spread of the disease and increase the chance of survival. Our patient had early serial debridement done, and which may have been the keys to success in avoiding wide spread of the infection. The extent of the skin debridement at the early stage is very difficult because the skin may often appear normal, though when investigated microscopically, the normal-appearing soft tissue showed extensive vascular microthromboses as well as vasculitis. This finding indicated that this tissue which has a normal external appearance has a high risk of full thickness necrosis [9, 10].

There was no need for any reconstruction of the chest wall of the patient unlike most of the reported cases of chest wall necrotizing fasciitis where reconstruction has been either by flaps or by skin grafts. Our patient is a neonate and most wounds at this age are forgiven with less scaring, which most likely prevented limitation in respiratory excursion.

The early multidisciplinary management of this patient may also have contributed to good outcome, that is, neonatologist, medical microbiologist, and plastic and chest surgeons.

The use of hyperbaric oxygen therapy was not applied in this case because we have no experience in using hyperbaric oxygen in a critically ill young patient, although its application is supported in the management of NF in some reports [7, 8].

In conclusion, NF is a life-threatening infection especially in the neonate in which early diagnosis with high index of suspicion, prompt resuscitation, aggressive surgical debridement, appropriate antibiotics therapy, and nutritional support with multidisciplinary approach will give a better outcome.
